# Do Miniature Eye Movements Affect Neurofeedback Training Performance? A Combined EEG-Eye Tracking Study

**DOI:** 10.1007/s10484-024-09625-6

**Published:** 2024-03-16

**Authors:** Silvia Erika Kober, Guilherme Wood, Sarah Schuster, Christof Körner

**Affiliations:** 1 Department of Psychology, University of Graz, Universitaetsplatz 2/III, 8010 Graz, Austria; 2 BioTechMed-Graz, Graz, Austria

**Keywords:** Eye tracking, SMR, Gamma, Neurofeedback, Miniature saccades, Responder

## Abstract

EEG-based neurofeedback is a prominent method to modulate one’s own brain activity in a desired direction. However, the EEG signal can be disturbed by artifacts, e.g., eye movements, which can consequently confound the neurofeedback performance. Involuntary miniature eye movements can be hardly detected by conventional EEG correction methods such as recording the electro-oculogram (EOG) and subtracting EOG activity from the EEG signal. However, such miniature eye movements can influence EEG activity, especially in the Gamma frequency range, enormously. In the present study, we investigated whether power in different EEG frequencies can be effectively modulated by self-control of brain signals during neurofeedback training and/or whether changes in EEG power are provoked by miniature eye movements during the training. To this end, 24 participants performed one session of SMR and one session of Gamma neurofeedback training. Additionally, in each training session sham feedback was performed. An eye tracker was used to detect miniature eye movements (< 1°) during neurofeedback training. About two thirds of the participants were able to increase their SMR power over the course of NF training, while one third was able to increase Gamma power. Generally, miniature eye movements induced a strong Gamma power increase. The number of eye movements also increased numerically over the course of the NF training. However, we did not find a significant relationship with the NF training performance. This is a first indication that miniature saccades do not affect NF training performance, but should not be neglected during NF training. Our results have to be confirmed in future studies.

**Supplementary Information:**

The online version contains supplementary material available at 10.1007/s10484-024-09625-6.

## Introduction

Neurofeedback (NF) enables voluntary control over one’s own brain activity through real-time feedback of that activity. The successful modulation of brain signals during NF training has been associated with improvements in cognitive and motor functions, and it may influence behaviour and affective states (Gruzelier, 2014; Kropotov, 2009; Wolpaw et al., 2002). Most NF studies used electrical brain activity measured by the electroencephalogram (EEG) as feedback signal (Enriquez-Geppert et al., 2017; Gruzelier, 2014; Kropotov, 2009). EEG has many advantages such as a high temporal resolution and the availability of cost-effective portable systems. However, compared to other neuroimaging techniques (e.g., near-infrared spectroscopy NIRS), EEG is very susceptible to artifacts (Enriquez-Geppert et al., 2017; Ninaus et al., 2014). Especially, eye and muscle artifacts are most common. These artifacts generate activity affecting the whole EEG power spectrum including EEG frequencies, which are used as feedback frequencies during NF training (Enriquez-Geppert et al., 2017). If these artifacts are not controlled during NF training, they may interfere with the effect of NF training. For instance, NF users may falsely learn to modulate their eye movements to influence the EEG feedback during NF training rather than the target EEG feedback frequency band directly (Enriquez-Geppert et al., 2017).

Muscle artifacts caused by large body movements or muscle tension generally produce noisy signals with high frequencies and a large amplitude, which are easier to detect in the raw EEG than eye movements. Larger eye movements such as blinks can also be detected and removed from the raw EEG, for instance by using regression methods, when the electro-oculogram (EOG) is recorded simultaneously (Jiang et al., 2019; Kobler et al., 2017). However, smaller eye movements, such as very small saccades, cannot be fully detected and removed using standard methods such as recording exogenous reference channels (i.e., EOG) and subtraction of the estimated artifacts from the EEG (Dimigen, 2020; Jiang et al., 2019). In the present study, we used eye tracking to detect small miniature eye movements (saccades) during NF training to investigate their possible effects on NF training performance.

Miniature saccades can elicit an increase of EEG activity in a broad Gamma (30–90 Hz) frequency range (Yuval-Greenberg et al., 2008), but slower EEG frequencies such as Alpha (8–12 Hz) activity can also be influenced by miniature saccades (Liu et al., 2023). Such miniature saccades or micro saccades are small saccades with an amplitude of typically 1° visual angle (v.a.) or less and an occurrence of approximately 0.5–2.0 Hz (Martinez-Conde et al., 2009). They occur involuntarily during fixation and are involved in a number of aspects of visual perception such as the perception of apparent motion (Laubrock et al., 2008) or the counteracting of visual fading during perception (Martinez-Conde et al., 2006). They also play an important role in attention and cognition; for instance, they can indicate the direction of covert attention (Engbert & Kliegl, 2003), and their rate changes with the type of stimulus in the oddball task (Valsecchi et al., 2007).

In a combined EEG-eye tracking study, Yuval-Greenberg et al. (2008) showed that EEG Gamma power increases time-locked to the onset of involuntary miniature eye movements. This saccade-related increase in EEG Gamma activity reflects the so-called saccadic “spike potential” (Keren et al., 2010). If the presence of (miniature) saccades varies systematically with experimental conditions, their activity can be misinterpreted as genuine brain activity elicited by experimental manipulations. The finding of Yuval-Greenberg et al. was replicated in many different combined EEG-eye tracking studies (e.g., Dimigen & Ehinger, 2021; Katz et al., 2020; Plöchl et al., 2012). Thus, there is strong evidence that miniature saccades affect a broad EEG frequency range.

In the present study, we used an established NF paradigm (Enriquez-Geppert et al., 2017; Kober et al., 2015, 2017b) to investigate the effects of miniature saccades on NF performance. We used two EEG feedback frequencies: SMR (sensorimotor rhythm, 12–15 Hz), since SMR-based NF training is one of the most evaluated and established NF trainings (Gruzelier, 2014; Kober et al., 2015; Kropotov, 2009), and Gamma (36–44 Hz), since miniature saccades should affect EEG frequencies particularly in this frequency range (Yuval-Greenberg et al., 2008).

A large body of literature provides evidence that a proportion of NF users is not able to voluntarily modulate their own brain activity during NF training (Allison & Neuper, 2010; Autenrieth et al., 2020; Weber et al., 2020). These users are called non-responders. The percentage of non-responders seems to depend on the NF paradigm. In SMR-based NF studies, about 30% of NF users are non-responders (Kober et al., 2017b, 2017c), while the number of non-responders seems to be higher in Gamma-based NF training studies (Kober et al., 2013, 2017b). But there are also SMR-based NF studies reporting higher rates of non-responders. For instance, Veilahti et al. (2021) classified 60% of adults with ADHD receiving SMR NF training as “non-learners”. Studies in which the NF learning rates were examined with other NF protocols, e.g., frontal Alpha (8–12 Hz) NF, even report a non-responder rate of up to 75% (Davelaar et al., 2018). In the present study, we splitted participants into groups of responders and non-responders based on their NF training performance to reveal possible differences in miniature saccadic activity and related induced Gamma activity between those groups. Additionally, we compared the effects of real and sham feedback conditions to investigate specific and unspecific effects of NF training (Ros et al., 2020; Thibault et al., 2017).

The aim of the present study was to document the effects of saccades, especially miniature saccades, on NF training performance. Hence, we did not use the eye tracking data to remove the influence of saccades from the EEG signal but rather to relate eye movements to specific characteristics of the EEG data in a combined analysis. In the practical application of NF training, a simultaneous recording of EEG and eye tracking data is hardly possible or feasible. Online artifact removal during NF training is also hardly possible in clinical practice or not implemented in hitherto commercially available NF training systems. Therefore, our aim is to investigate whether the ability to modulate one’s own EEG activity in a desired direction during NF training is affected by miniature saccades or not, which will have practical implications for the interpretation of NF training success.

## Methods

### Participants

Twenty-four healthy young adults (mean age 23.17 years, *SD* = 3.60, 19 females) participated in this study. All volunteers had normal or corrected-to-normal vision (wearing contact lenses) and were not deuteranopic. All participants performed under all experimental conditions (SMR real and sham feedback conditions, Gamma real and sham feedback conditions) and were blind to these experimental conditions. SMR and Gamma NF was performed on different days. For the Gamma NF, three participants had to be excluded from the final data analysis due to excessive EEG artifacts.

Participants fulfilled the following inclusion criteria: no neurological, psychological or other severe diseases, no reflex epilepsy, in which external stimuli such as visual stimuli (visual feedback screen, moving feedback objects) can trigger an epileptic seizure, no skin problems or wounds in the head area, which might jeopardize EEG recordings, no medication that affects the central nervous system. Participants gave written informed consent before the start of the measurement. For their participation they received either research credit hours for their Psychology Bachelor program or money (50€ in total). The ethics committee of the University of Graz, Austria, approved all aspects of the present study in accordance with the Declaration of Helsinki (GZ. 39/29/63 ex 2016/17).

After data assessment, participants were assigned to a responder and a non-responder group based on their NF performance (see results section for details about this classification), separately for the SMR and Gamma NF condition. Table 1 summarizes how many participants were assigned to the responder and non-responder group for SMR and Gamma NF.

**Table 1:** Assignment of participants to the responder and non-responder groups according to their NF training performance, separately for the SMR and Gamma NF training condition

	SMR NF	Gamma NF
Sample size N	24	21
Number of responders (number of females)	14 (10)	8 (6)
Number of non-responders (number of females)	10 (9)	13 (10)
Mean age in years of responders (SD)	23.29 (3.36)	22.13 (2.75)
Mean age in years of non-responders (SD)	23.00 (4.08)	24.31 (4.05)

The study used a within-subject design

### Design and Neurofeedback Training

The experimental design and the outcome of the present NF study are reported in line with the Consensus on the Reporting and Experimental Design of clinical and cognitive-behavioural Neurofeedback studies (CRED-nf) (Ros et al., 2020). The CRED-nf checklist can be found in the supplementary material B.

All participants performed a SMR- and a Gamma-based NF training session on two different days (half of the participants started with SMR, the other half with Gamma NF training). In each session, a real and a sham feedback condition were conducted. The order of real and sham feedback was also balanced across participants. Both the real and the sham feedback condition included one baseline run of 3 min (in which participants should only watch the feedback bars without trying to control them) and 6 feedback runs (in which participants were asked to actively control the feedback bars) with a duration of 3 min each. Between these 3-min runs, short breaks with a variable duration were integrated. For the SMR NF training, Cz was used as feedback electrode. For the Gamma NF training, POz was used as feedback electrode. Online processing of the EEG raw signal included a band-pass filter in the respective target frequency bands (6th order Butterworth IIR filter) and squaring the filtered data to obtain power estimates. To generate smooth visual feedback, a moving average of 256 samples was applied and updated on the computer screen at a rate of 10 Hz.

During the NF training, participants were presented with three blue bars (RGB 0,0,255) on a black background (RGB 0,0,0). The bars represented EEG power in three frequency bands. The bar in the middle of the screen depicted SMR (12–15 Hz) power for the SMR NF training and Gamma (36–44 Hz) power for the Gamma NF training. It was ca. 11.0° wide and it changed vertically in size whenever SMR or Gamma power increased or decreased, respectively. To the left and right of the central bar two additional bars were presented depicting EOG artefacts (4–7 Hz, control frequency I) and muscle artefacts (50–100 Hz, control frequency II), respectively (Doppelmayr & Weber, 2011; Weber et al., 2011). They were both ca. 3.1° wide. These control bars were the same in the SMR and Gamma NF training.

**Fig. 1:**
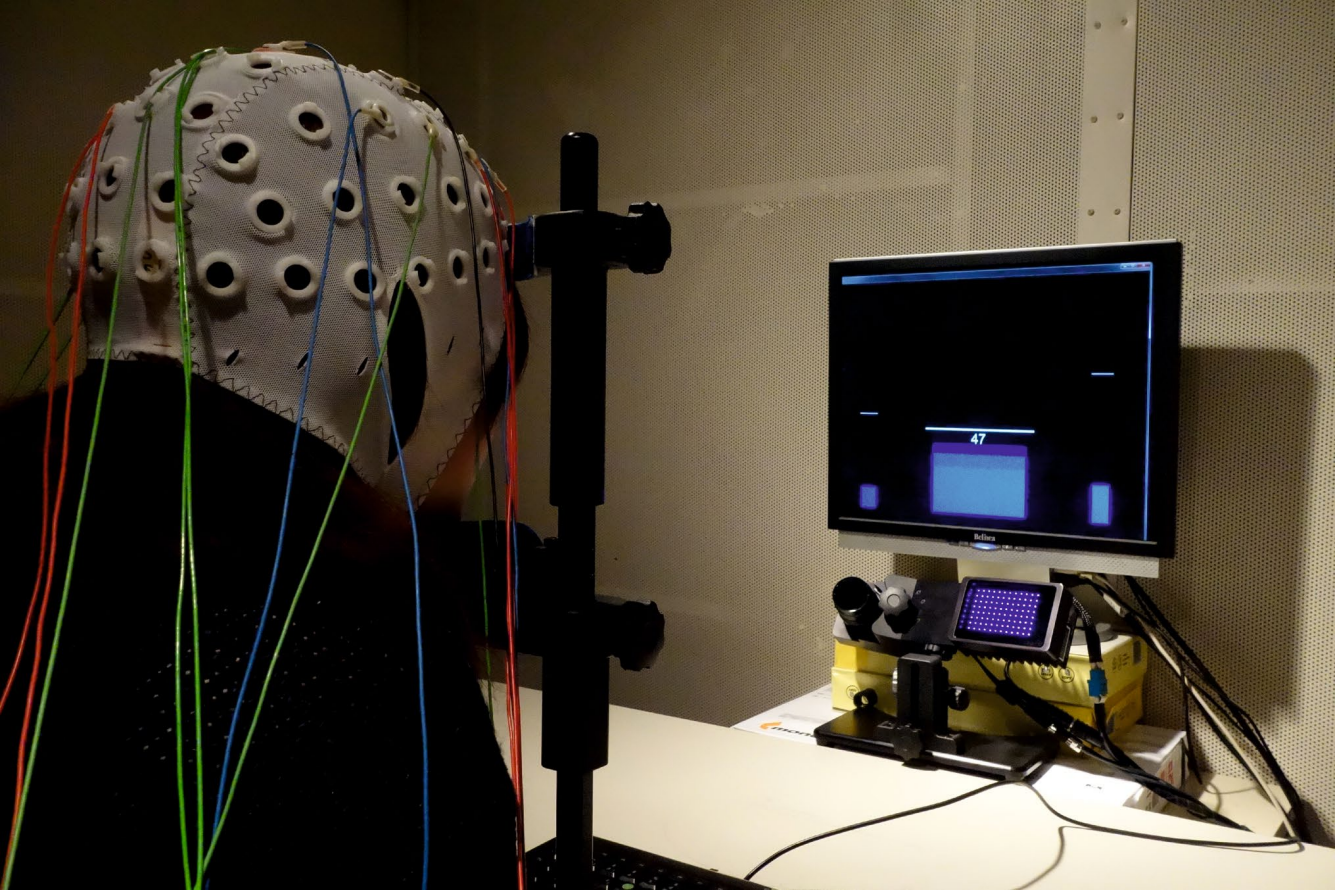
Experimental setup. The eye tracker was placed under the feedback screen displaying three vertically moving feedback bars, their threshold lines, and the reward points. Participants’ head movements were stabilized by a chin and forehead support during EEG recording

The baseline run at the beginning of the NF training session was used to calculate individual thresholds for each bar. The median of the SMR or Gamma power and the median + 1.0 *SD* of the control frequencies (4–7 Hz and 50–100 Hz), respectively, were used as threshold values (drawn as horizontal white lines over the feedback bars, see Fig. 1) for the first feedback run. The SMR and Gamma thresholds were adapted after each feedback run based on the median power of the immediately preceding run.

Participants were instructed to increase the height of the bar in the middle of the screen beyond the predefined threshold line while keeping the bars on the left and right side of the screen constantly below their threshold lines during the feedback runs. If successful in doing so, they received reward points displayed in white (RGB 255,255,255) with a height of ca. 2.7° placed below the white threshold line of the middle bar. The reward points were incremented by one unit each time the target state was achieved for 250 ms. If the control bars (4–7 Hz and 50–100 Hz) were above their predefined thresholds, the color of these bars changed from blue to red (RGB 128,0,0). In all NF conditions, participants received the minimal instruction of being physically relaxed and mentally focused during the feedback runs.

In both sham and real feedback conditions, the movement of the control bars depicted participants’ real 4–7 Hz and 50–100 Hz power in real-time. In the real feedback condition, the central bar depicted the SMR or Gamma power of the participants in real-time, respectively. In the sham feedback condition, the movement of the central bar depicted a previously recorded SMR or Gamma power training of an unrelated person. Because in the sham condition the feedback on the control frequencies (4–7 Hz and 50–100 Hz) was genuine as in the real feedback condition, the subjective feeling of control was the same in both conditions.

### Questionnaires

To investigate possible differences in psychological constructs such as control beliefs, motivation, subjectively perceived level of concentration during NF training, mental strategies used during NF training, participants filled out respective questionnaires at the beginning of the first session. We did not find any such differences. A description of the questionnaires, the detailed results per group and statistical comparison can be found in the supplementary material A.

### EEG Recording

We recorded EEG with 24 electrodes using two 16 channel g.USBamp standard amplifiers (g.tec, Graz, Austria). A linked mastoid reference was used, the ground was placed at C5. The following EEG channels were recorded: AFz, F7, F3, Fz, F4, F8, FCz, T7, C3, C1, Cz, C2, C4, T8, CPz, P7, P3, Pz, P4, P8, POz, O1, Oz, and O3. For the NF training, only Cz (for SMR NF training) and POz (for Gamma NF training) were used. Vertical and horizontal EOGs were recorded with three electrodes in total, two were placed on the outer canthi of the eyes and one was placed superior to the nasion. Electrode impedances were kept below 5 kΩ for the EEG recording and below 10 kΩ for the EOG recording. EEG signals were digitized at 256 Hz.

### Eye-Movement Recording

We used an EyeLink 1000 eye tracker (SR Research, Canada) with a desktop mount (remote) setup to record eye movements. Participants’ head movements were stabilized by a chin and forehead rest (Fig. 1). Eye movements were recorded monocularly with 500 Hz sampling rate. The distance from the eye tracker camera to the left eye of the participants was 55 cm. The distance from the feedback screen to the participants’ eyes was 64 cm. The eye tracker was calibrated before each 3-min run. The average deviation between calibration and validation was less than 1° v.a. in all cases. The velocity threshold for saccade detection was 30°/sec, the acceleration threshold was 8000°/sec^2^. The procedure was controlled by a presentation computer attached to the eye tracker via an Ethernet connection. This machine ran Matlab R2015B (The MathWorks Inc, Natick, MA, USA) interfacing with Simulink software (The MathWorks Inc, Natick, MA, USA). We used the Psychophysics and Eyelink Toolbox extensions (Brainard, 1997; Cornelissen et al., 2002; Kleiner et al., 2007; Pelli, 1997) to control the eye tracker. Visual feedback was presented on a Belinea 19-inch TFT monitor with a native resolution of 1280 × 1024 pixels at 60 Hz.

### Analysis of EEG and Eye Movement Data

To synchronize EEG and eye tracking data, triggers were used as recommended by Dimigen et al. (2011). A trigger was sent every 10 s from the presentation PC to the eye tracker and the EEG recording computer. In addition, a start and an end trigger were sent at the beginning and end of each run, respectively. The eye tracking data between the start and end triggers were down-sampled to the sampling rate of the EEG.

Data were processed offline with MATLAB R2020b (The MathWorks Inc, Natick, MA, USA) and the EEGLAB toolbox 2022.0 (Delorme & Makeig, 2004), with the EYE-EEG 0.41 (Dimigen et al., 2011) toolbox. The EYE-EEG toolbox was used for simultaneous analysis of EEG and eye tracking data. EEG and eye tracking data were imported into EEGLAB and synchronized based on the common online triggers using the EYE-EEG plugin.

The EYE-EEG automatic correction procedure was used in which eye tracking data are used to identify ocular artifacts (e.g., blinks), and segments containing such artifacts were removed. Saccades and fixations were automatically detected using the velocity-based detection algorithm (Engbert & Mergenthaler, 2006) provided with the EYE-EEG toolbox. For this offline saccade detection, the minimum saccade duration was set to two samples (minimum duration of the (micro)saccade: 4 ms, velocity threshold = 6 times the median velocity and the minimum inter-saccadic interval = 50 ms). Fixations were defined as intervals between saccades. Finally, a manual artifact correction was performed to remove further artifacts (e.g., muscle artifacts). Artifact rejection was performed by a trained EEG expert. In sum, 25% of the data had to be excluded due to artifacts.

For the analysis of SMR-based NF training data, absolute power values in the SMR (12–15 Hz) and the control (4–7 Hz and 50–100 Hz) frequency range over Cz were extracted and averaged per run, while for the analysis of Gamma-based NF training data, Gamma (36–44 Hz) and the control frequencies (4–7 Hz and 50–100 Hz) over POz were extracted and averaged per run. Additionally, ERDS (event-related desynchronization / synchronization) values in a broader Gamma frequency range (30–90 Hz) were analyzed over POz, time-locked to the onset of saccades (Pfurtscheller & Lopes da Silva, 1999). ERDS values reflect the percentage change in EEG power between a baseline interval (100 ms before the onset of a saccade) and an active interval (100 ms after the onset of a saccade). An increase in power from baseline to task is reflected in a synchronization (ERS).

### Statistical Analysis

To test whether power in the broad Gamma frequency range significantly increased after a miniature saccade compared to the baseline interval, as shown by Yuval-Greenberg et al. (2008), one-sided *t*-tests against zero were performed for the dependent variable Gamma ERDS.

To classify participants in responders and non-responders based on their NF performance, regression analyses were performed per participant with feedback run number (6 feedback runs) as predictor variable and either SMR or Gamma power as dependent variable. Hence, we obtained a regression slope for each individual per condition indicating whether EEG power increased (positive regression slope), decreased (negative regression slope), or remained stable (slope = 0) in each individual participant across the six feedback runs. One-sample *t*-tests were calculated for each condition to test whether the regression slopes differ from zero to verify the consistency of the learning effects.

For a first assessment whether the classification in NF responders and non-responders was related to the number of miniature saccades, we also calculated regression slopes (with feedback run number as predictor variable and the number of miniature saccades per second as dependent variable) per group (NF responders and non-responders) and condition (real/sham SMR and Gamma feedback) for the miniature saccades as dependent variable. One-sample *t*-tests were calculated per group (NF responders and NF non-responders) and condition (SMR and Gamma NF, real and sham feedback) to test whether these regression slopes differ from zero.

We performed correlation analyses between regression slopes of EEG power (NF performance) and regression slopes of miniature saccades per group (NF responders and NF non-responders) and condition (SMR and Gamma feedback). For all analyses, alpha level was set to *p* = 0.05 and adjusted for multiple post-tests using Bonferroni correction.

In a final step, we performed a second classification of participants into a group with an increasing number of miniature eye movements across the NF training runs (saccade increasers) versus a group with non-increasing miniature eye movements (saccade non-increasers). Therefore, regression analyses were performed with feedback run number (6 feedback runs) as predictor variable and the number of miniature saccades per second as dependent variable. Participants with a positive regression slope were assigned to the saccade increaser group, participants with a zero or negative regression slope were assigned to the saccade non-increaser group. Then we statistically compared the observed and expected relative frequencies of being a NF responders/non-responders and saccade increasers/non-increasers using Chi-Square tests. Additionally, we tested whether SMR or Gamma power across NF runs (slopes) differed from zero using one-sample *t*-tests separately for saccade increasers and non-increasers.

## Results

### Induced Gamma Activity After Miniature Saccades

**Fig. 2:**
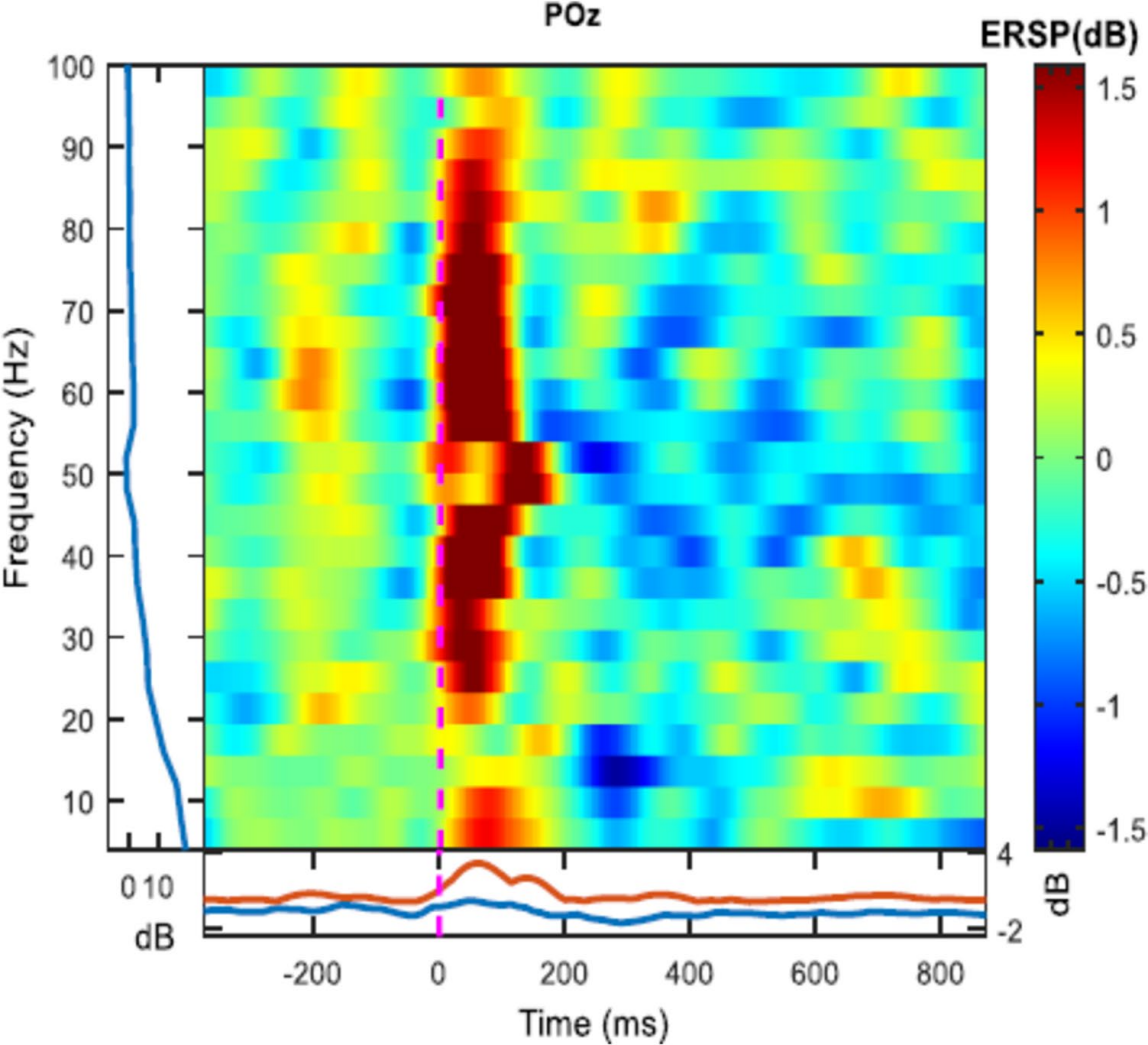
Representative example of a time–frequency plot for one subject showing a broad Gamma power increase at location POz after saccade onset (time point 0), averaged across all miniature saccades during NF training. ERSP: Event-Related Spectral Perturbation

In a first step, we wanted to check whether miniature saccades induced Gamma activity in a broader frequency range (30–90 Hz). Therefore, we calculated saccade-related power changes in this broader Gamma frequency range. The resulting ERDS values reflect the percentage change in EEG power between a baseline interval (100 ms before the onset of a saccade) and an active interval (100 ms after the onset of a saccade). An ERS reflects an increase in Gamma power from baseline to an active interval. Figure 2 shows an example of such a saccade-induced Gamma power increase.

To test whether Gamma power significantly increased after a miniature saccade compared to the baseline interval, one-sided *t*-tests against zero were performed comparing averaged ERDS values during the active interval with 0, since the ERDS baseline interval is on average 0. This series of *t*-tests showed a significant increase in Gamma ERS compared to the baseline in all conditions (Table 2). Hence, Gamma power significantly increased after the occurrence of a miniature saccade.

**Table 2:** Results of one-sample *t*-tests comparing Gamma ERDS values against 0 after saccade onset

	*df*	*t*-value	*p*-value
SMR NF real feedback	21	4.918	0.000036*
SMR NF sham feedback	21	5.785	0.000005*
Gamma NF real feedback	20	5.075	0.000029*
Gamma NF sham feedback	20	4.846	0.000049*

*Asterisks indicate significant results

### NF Performance—Classification into Responders and Non-responders

**Fig. 3:**
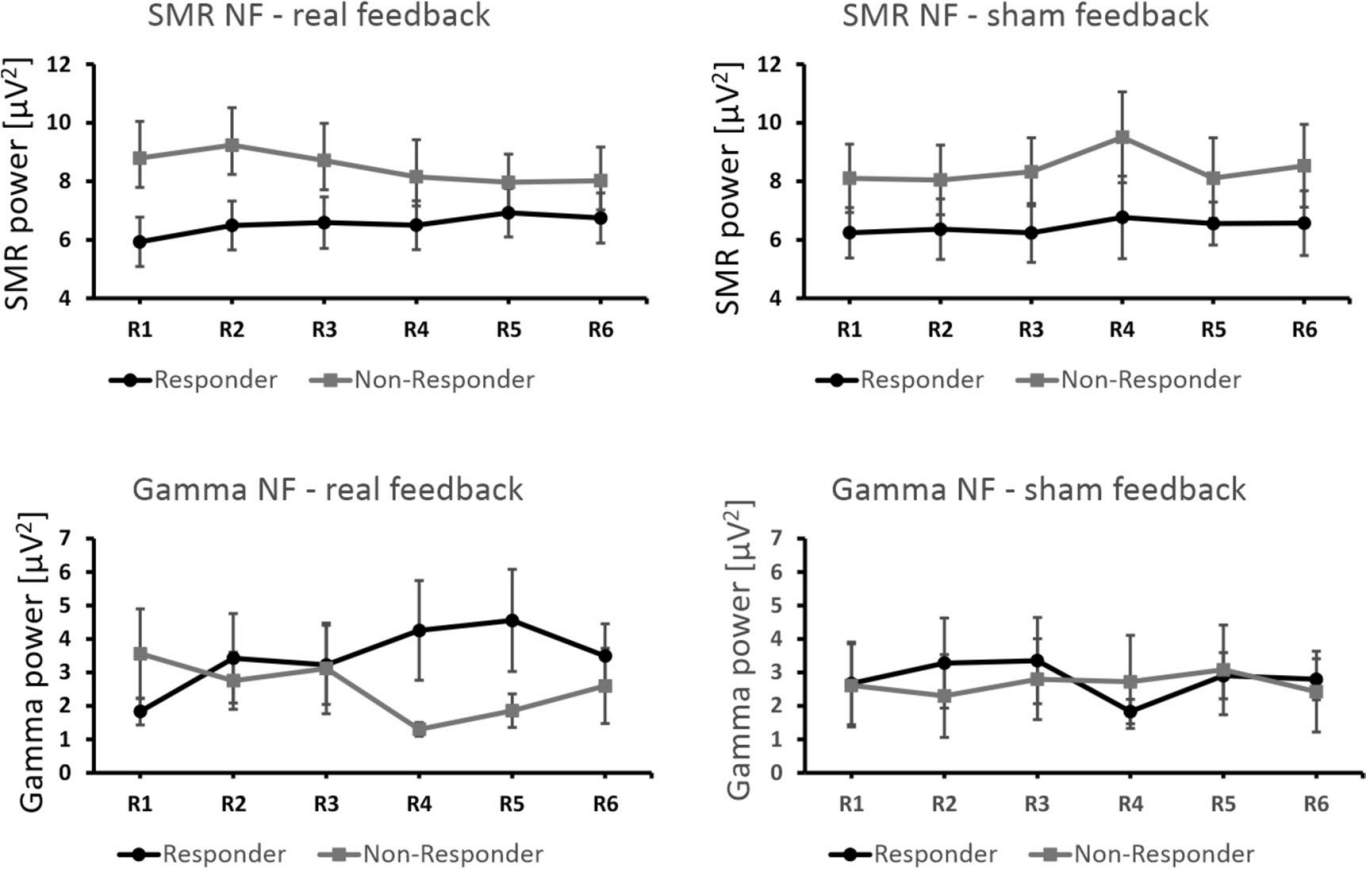
Changes in EEG feedback frequencies (mean SMR power top panels, mean Gamma power bottom panels; real feedback left panels, sham feedback right panels) across feedback runs R1–R6, presented separately for responders and non-responders. Error bars show *SEM*

Next, we analyzed the NF training data in detail. Based on their NF performance, participants were assigned to a responder and to a non-responder group. Participants who were able to increase the power in the target EEG feedback frequency (either SMR or Gamma, respectively) across real feedback runs were assigned to the responder group. Participants who were not able to increase power in the EEG feedback frequency across real feedback runs were assigned to the non-responder group. For the respective assignment, regression analyses were performed for each participant with feedback run number (6 feedback runs) as predictor variable and either SMR or Gamma power as dependent variable. Participants with a positive regression slope were assigned to the responder group, participants with a zero or negative regression slope were assigned to the non-responder group. This kind of classification is a standard procedure in line with previous NF training studies (e.g., Kober et al., 2019). The group assignment was performed separately for real SMR- and Gamma-based NF. Hence, a participant could be a responder in one NF protocol (e.g., SMR-based NF) and a non-responder in the other NF protocol (e.g., Gamma-based NF). If a participant was identified as a responder based on the real feedback SMR NF data, this participant was also assigned to the responder group for the SMR sham feedback condition. The same applies to Gamma-based NF training. The classification into responder and non-responder was based on the changes in Gamma power during the real feedback condition. If a participant was assigned to the responder group based on the real feedback Gamma activity, this participant was also a responder in the Gamma sham feedback condition. For SMR-based NF, 14 participants showed a regression slope > 0 and thus were assigned to the responder group (see Table 1). The one-sample *t*-tests against zero showed that the positive regression slopes of the responder group as well as the negative regression slopes of the non-responder group differed significantly from zero during real SMR feedback but not during sham feedback (Table 3). For Gamma-based NF, 8 participants showed a regression slope > 0 and thus were assigned to the responder group (see also Table 1). The positive regression slope of the responder group during real Gamma-based NF differed significantly from zero. However, this effect was not significant any more after Bonferroni correction (Table 3). Seven participants were in the non-responder group for both SMR and Gamma-based NF training, five participants were in the responder group for both SMR and Gamma-based NF training. This indicates that a successful increase in SMR power during NF training does not entail a successful NF performance during Gamma-based NF training and vice versa. Figure 3 shows average power changes of responders and non-responders across NF training runs for real and sham feedback as well as for SMR- and Gamma-based NF, respectively. When receiving real feedback, responders showed a linear increase in the EEG feedback frequency (either SMR or Gamma) while non-responders showed a decrease across feedback runs (see Fig. 3, left). Sham feedback led to no prominent changes in EEG power for either group (see Fig. 3, right). As can be seen in Fig. 3, the non-responders receiving real SMR feedback started out at higher power values compared to the responders. SMR power decreased for the former group and increased for the latter group over time. To rule out the possibility that such effects were due to regression to the mean, we conducted a further classification into responders and non-responders based on the sham data. This random division of the groups did not yield any results indicating regression to the mean.

**Table 3:** Results of one-sample *t*-tests comparing regression slopes of SMR/Gamma power against 0

	*df*	*t*-value	*p*-value
SMR NF real feedback—responders	13	4.74	0.000389*
SMR NF real feedback—non-responders	9	− 3.81	0.004152*
SMR NF sham feedback—responders	13	1.95	0.073
SMR NF sham feedback—non-responders	9	1.86	0.096
Gamma NF real feedback—responders	7	2.63	0.034
Gamma NF real feedback—non-responders	12	− 1.57	0.142
Gamma NF sham feedback—responders	7	− 0.18	0.864
Gamma NF sham feedback—non-responders	12	0.84	0.419

*Asterisks indicate significant results

### Miniature Saccades for NF Responders and Non-responders

The saccades observed during NF training were relatively small in amplitude. Seventy-eight percent of all saccades were smaller than 3° v.a. and 50% were smaller than 1° v.a. (see Figure D1 in the supplementary material D). This indicated that most of the saccades occurred during fixation of one of the three feedback bars, and that longer saccades occurred only occasionally, for example when subjects moved their eyes between the outer feedback bars. Following Yuval-Greenberg et al. (2008), we therefore focused on ‘‘miniature saccades’’, which were defined as saccades smaller than 1° v.a..

**Fig. 4:**
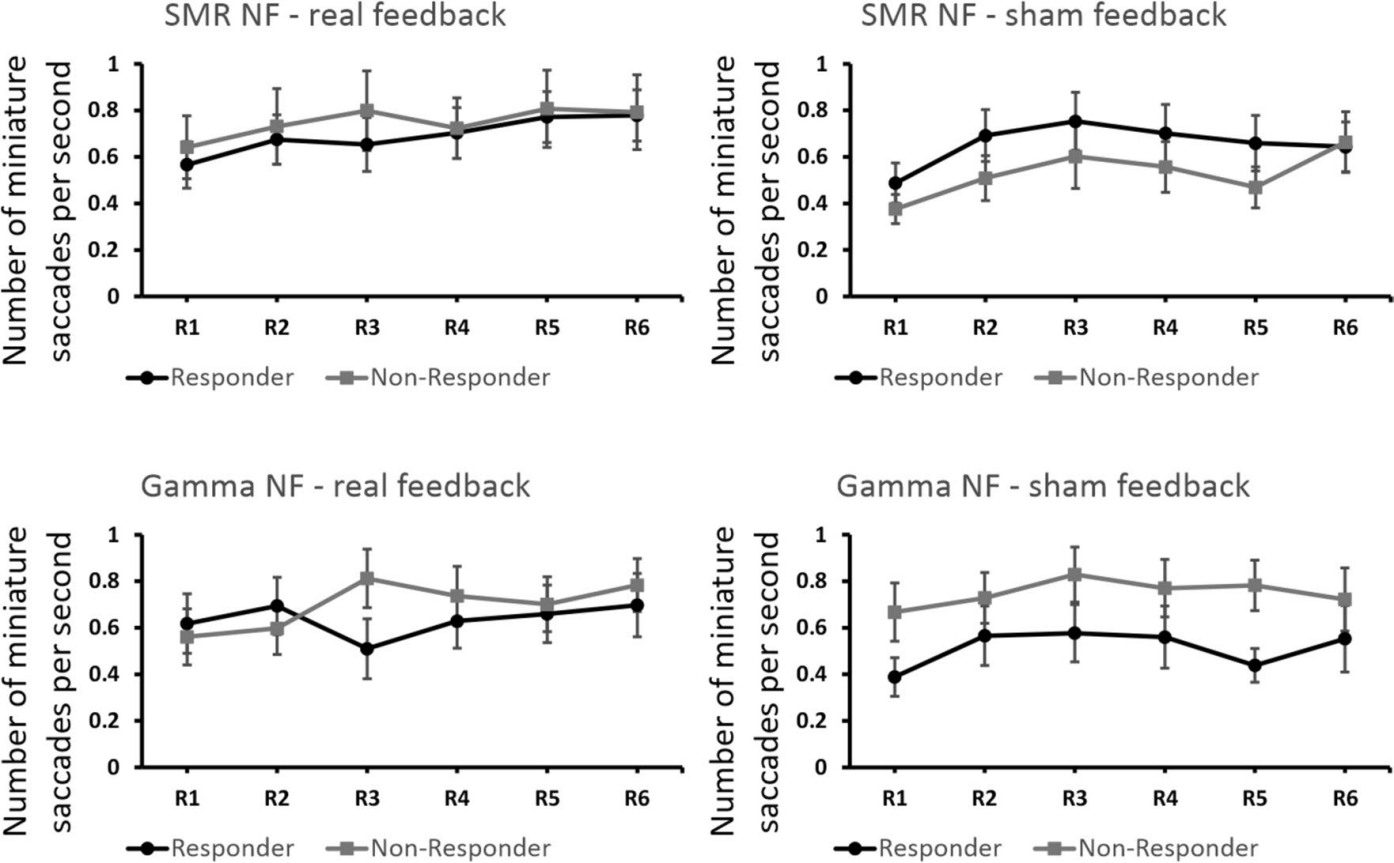
Changes in the number of miniature saccades per second across feedback runs R1–R6, presented separately for responders and non-responders and for the different conditions (SMR NF top panels, Gamma NF bottom panels; real feedback left panels, sham feedback right panels). Error bars show *SEM*

Similar to the analysis of the NF training data, we determined regression slopes for each condition and NF group (using feedback run number as predictor variable) for the miniature saccades per second as dependent variable (Fig. 4). One-sample *t*-tests were calculated per group (NF responders and NF non-responders) and condition (SMR and Gamma NF, real and sham feedback) to test whether the regression slopes differed from zero. Note that for SMR-based NF one participant of the responder and one participant of the non-responder group did not have useable eye tracking data. The *t*-tests revealed no significant results (Table 4). Only responders during real SMR feedback showed a trend (*p* < 0.10) towards a positive slope for miniature saccades over the feedback runs (Fig. 4).

**Table 4:** Results of one-sample *t*-tests comparing regression slopes of miniature saccades against 0

	*df*	*t*-value	*p*-value
SMR NF real feedback—responders	12	2.04	0.064
SMR NF real feedback—non-responders	8	1.53	0.165
SMR NF sham feedback—responders	12	0.97	0.350
SMR NF sham feedback—non-responders	8	1.86	0.100
Gamma NF real feedback—responders	7	0.58	0.583
Gamma NF real feedback—non-responders	12	1.58	0.141
Gamma NF sham feedback—responders	7	0.89	0.401
Gamma NF sham feedback—non-responders	12	0.65	0.531

*Asterisks indicate significant results

Changes in the number of all saccades (larger and smaller than 1° v.a.) across the feedback runs can be found in the supplementary material D.

### Correlation Between NF Performance and Miniature Saccades

To explore the relation between NF performance and miniature saccades more directly, we correlated the NF slopes with the slopes of the miniature saccades across feedback runs to reveal a potential relationship. We computed these correlations across all participants as well as for the responder and non-responder groups, respectively. To reduce the number of correlations, we computed these correlations only for the real feedback conditions.

All correlations were positive but not significant (Table 5). During real Gamma NF, the non-responders showed a trend (*p* < 0.10) towards a significant positive relationship between the NF and miniature saccades slopes.

**Table 5:** Correlation coefficients *r* (*p*-value) for NF slopes and slopes of miniature saccades across feedback runs

	All participants	Responders	Non-responders
SMR NF real feedback	0.21 (0.34)	0.15 (0.61)	0.27 (0.49)
Gamma NF real feedback	0.27 (0.24)	0.16 (0.71)	0.49 (0.09)

### Miniature Saccades—Classification into Saccade Increasers and Non-increasers

In order to further investigate the relationship between miniature saccades and EEG activity, we also classified the participants into saccade increasers and non-increasers using regression analysis with feedback run number (6 feedback runs) as predictor variable and the number of miniature saccades per second during the real feedback conditions as dependent variable. Regression analyses were performed per participant. Participants with a positive regression slope were assigned to the saccade increaser group, participants with a zero or negative regression slope were assigned to the saccade non-increaser group. For SMR NF training, 17 participants were assigned to the saccade increaser group and 5 to the non-increaser group. For Gamma NF training, 14 participants were assigned to the saccade increaser group and 5 to the non-increaser group. Table 6 shows the observed and expected relative frequencies of NF responders/non-responders and saccade increasers/non-increasers per NF training. For example, in the sample of 22 participants with usable EEG and eye tracking data, there were 13 SMR NF responders (see Table 1, 14 participants were SMR NF responders, however, of these 14 participants, eye tracking data were only available for 13 participants); out of these 13, there were 10 participants who also were saccade increasers, resulting in an observed relative frequency of 10/22 ≈ 0.46. The expected frequency was calculated from the probability of being a NF responder/non-responder (relative observed frequency) * 0.5 (probability of being a saccade increaser/non-increaser was assumed to be 50%, i.e., chance); for example, 13/22 * 0.5 ≈ 0.30. A Chi-Square Test comparing observed and expected frequencies was significant neither for SMR NF training (*χ*^2^ (1) = 0.30, *p* = 0.59) nor for Gamma NF training (*χ*^2^ (1) = 0.24, *p* = 0.62). This result demonstrates that the observed proportions of participants who were classified as NF responders/non-responders and saccade increasers/non-increasers did not deviate from chance expectation.

**Table 6:** Observed (expected) relative frequencies of NF responders/non-responders and saccade increasers/non-increasers, presented separately for real SMR NF and real Gamma NF

	Saccade increasers	Saccade non-increasers
SMR NF real feedback		
NF responders	0.46 (0.30)	0.14 (0.30)
NF non-responders	0.32 (0.20)	0.09 (0.20)
Gamma NF real feedback		
NF responders	0.26 (0.16)	0.05 (0.16)
NF non-responders	0.47 (0.34)	0.21 (0.34)

**Fig. 5:**
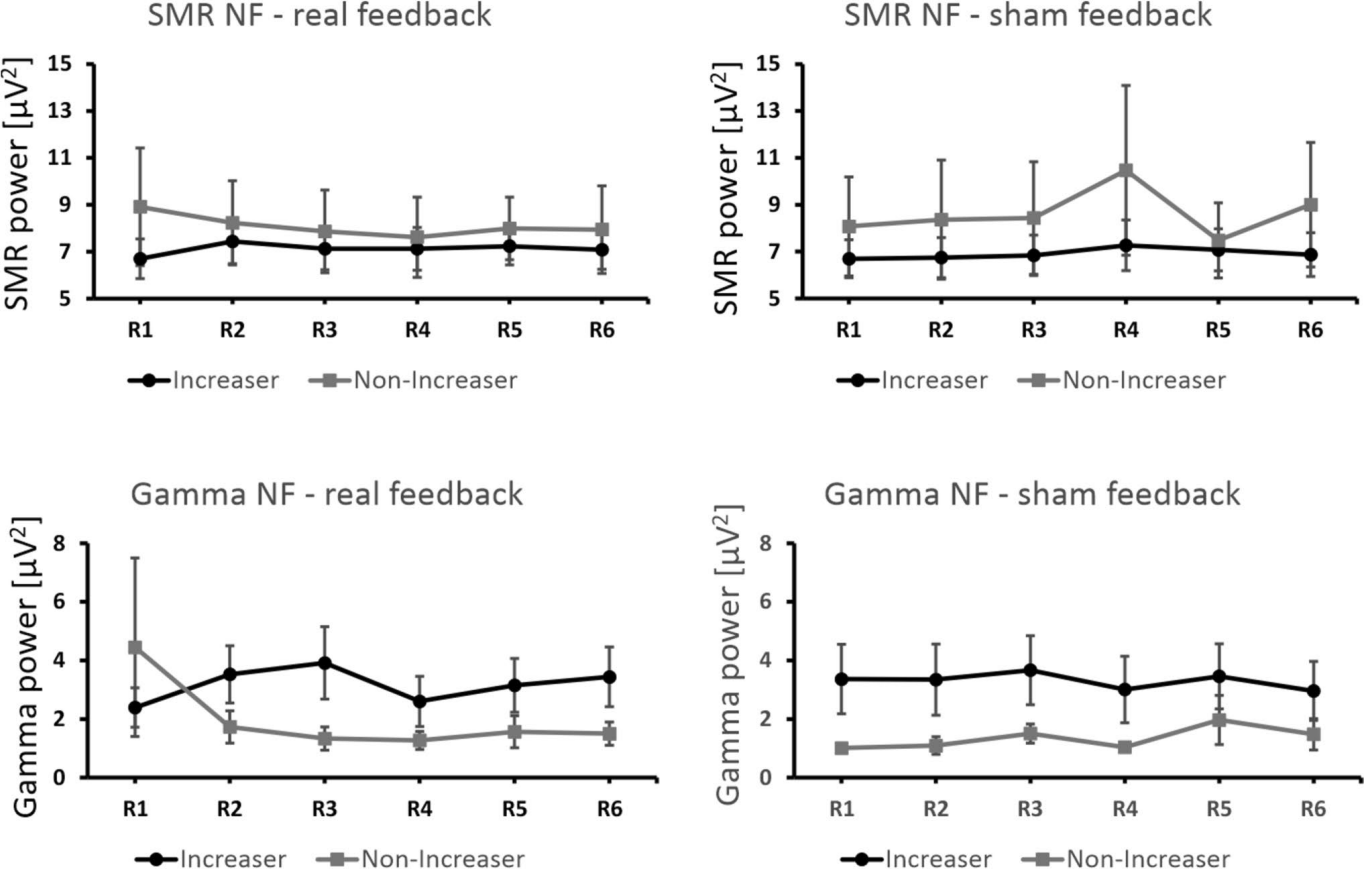
Changes in EEG feedback frequencies (SMR power top panels, Gamma power bottom panels; real feedback left panels, sham feedback right panels) across feedback runs R1–R6, presented separately for saccade increasers and non-increasers. Error bars show *SEM*

In a final analysis, we related the saccade increaser/non-increaser classification to the NF performance directly. Figure 5 shows changes in SMR and Gamma power across NF runs for saccade increasers and non-increasers. When comparing the regression slopes (regression analyses were performed for each participant with feedback run number as predictor variable and either SMR or Gamma power as dependent variable) per group (saccade increaser and non-increaser group) and condition (SMR/Gamma NF and real/sham feedback) against zero, no significant results could be observed (Table 7). This means that the classification based on saccadic behaviour was unrelated to NF performance.

**Table 7:** Results of one-sample *t*-tests comparing regression slopes of SMR/Gamma power against 0 for the saccade increasers/non-increasers groups

	*df*	*t*-value	*p*-value
SMR NF real feedback—saccade increasers	16	0.68	0.507
SMR NF real feedback—saccade non-increasers	4	− 0.47	0.664
SMR NF sham feedback—saccade increasers	16	1.99	0.064
SMR NF sham feedback—saccade non-increasers	4	2.50	0.067
Gamma NF real feedback—saccade increasers	13	0.84	0.416
Gamma NF real feedback—saccade non-increasers	4	− 1.05	0.354
Gamma NF sham feedback—saccade increasers	13	− 0.37	0.715
Gamma NF sham feedback—saccade non-increasers	4	1.21	0.294

## Discussion

In the present study, we investigated the effects of miniature eye movements on EEG-based NF performance. Participants’ eye movements were tracked during NF training in which they received visual feedback of either SMR (12–15 Hz) or Gamma (36–44 Hz) EEG power via three vertically moving bars. We focused our analysis on miniature saccades (saccades < 1° v.a.) because it is known that such miniature saccades elicit an increase in a broad EEG Gamma frequency range (Dimigen & Ehinger, 2021; Dimigen et al., 2011; Yuval-Greenberg et al., 2008). For this reason, we wanted to know whether these miniature saccades affect EEG-based NF training performance as well.

### Induced Gamma Activity After Miniature Saccades

In a first step, we checked whether we could replicate the findings by Yuval-Greenberg et al. (2008) whether miniature saccades induce Gamma activity in a broader frequency range (30–90 Hz). The analysis of event-related power changes in the broad Gamma frequency range (ERDS analysis) showed that miniature saccades led indeed to a significant increase in Gamma power after the onset of saccades.

### NF Performance

Participants were split up in responders and non-responders based on the change in EEG feedback frequency power (either SMR or Gamma, respectively) across the feedback runs (Kober et al., 2019). In SMR-based NF training, 58% of all participants were identified as responders. In Gamma-based NF training, only 38% of all participants receiving Gamma-based NF were classified as responders. This is in line with previous studies showing a higher rate of responders during one session of SMR-based NF training than during one session of Gamma-based NF training in healthy average individuals (Kober et al., 2013, 2017b, 2017c; Rubik, 2011). The reason for this difference in the number of responders and non-responders between NF protocols remains open and is beyond the scope of the present study. We refer to a number of studies trying to find predictors of NF training performance (Alkoby et al., 2017; Hammer et al., 2012; Kleih et al., 2010; Reichert et al., 2015; Weber et al., 2020; Witte et al., 2013).

For SMR-based NF training, responders receiving real feedback were indeed able to increase SMR power from early to late runs while non-responders—regardless of the feedback condition—were unable to achieve this. In the sham feedback condition, both responders and non-responders were not able to increase SMR power from early to late feedback runs. This result shows that real feedback led to a specific increase in SMR power while sham feedback did not, demonstrating that real SMR feedback led to successful NF training performance (Ros et al., 2020; Thibault et al., 2016). A further sign for the specificity and the success of the SMR-based NF training is that other EEG frequencies (control frequencies, 4–7 Hz and 50–100 Hz) did not change significantly during NF training (see supplementary material C) (Kober et al., 2017b).

During Gamma-based NF training, responders receiving real feedback showed the strongest increase in Gamma power across feedback runs. However, this effect was no longer significant after correcting for multiple comparisons. This again is in line with previous studies showing that participants are less successful in increasing Gamma power during NF than increasing the power of other EEG frequencies such as SMR (Kober et al., 2013, 2017b, 2017c; Rubik, 2011). Descriptively speaking, however, there was a trend comparable to SMR-based NF training. While responders showed an increase in Gamma power values from early to late runs when receiving real feedback, non-responders showed a decrease in Gamma power from early to late runs, and sham feedback led to either a decrease in Gamma power (responder group) or a constant Gamma power level (non-responder group) between early and late runs. Thus, responders showed an increase in either SMR or Gamma power, respectively, during real NF training, which is a sign of successful NF training performance.

As can be seen in Fig. 3, SMR power in the real and sham feedback condition was generally larger in the non-responder group than in the responder group, especially at the beginning of the NF training. A classification into responders and non-responders based on the sham data showed that the original classification was not due to regression to the mean. More importantly, such differences in SMR power at baseline cannot explain the differences in NF performance since prior studies showed that a higher baseline SMR power as found in the non-responder group is generally associated with a superior NF performance (Blankertz et al., 2010; Reichert et al., 2015).

### Number of Saccades for NF Responders/Non-responders

In the next step, we analyzed the eye tracking data to see how the number of saccades changed over the course of the NF training in NF responders and non-responders. It is important to point out that the participants in the present study were allowed to move their eyes freely across the feedback screen during NF training. From previous EEG studies it is known that such natural viewing conditions can increase task engagement (Welke & Vessel, 2022). A higher engagement in the NF task is beneficial for NF training performance, as there is evidence that letting one's thoughts digress during NF training to topics unrelated to the task can negatively affect NF performance (Kober et al., 2013).

The majority of saccades in our study were miniature saccades. A larger number of miniature saccades compared to regular saccades might indicate that participants fixated most of the time the reward points or the feedback bar in the middle of the screen. The smaller control bars on the left and on the right side of the screen that depicted artifact activity could be perceived peripherally. This could be the reason for the dominance of miniature saccades. Since we did not analyze directly where the participants were looking at, this is only speculative. However, for statistical analysis, we concentrated on miniature saccades.

While the *t*-tests against 0 revealed no significant changes in the number of miniature saccades per second across feedback runs, NF responders during real SMR feedback showed indeed a trend (*p* < 0.10) towards a positive slope of miniature saccades over the training runs (Fig. 4). Descriptively, all groups showed a numerically higher number of miniature saccades per second during the late NF runs compared to the early NF runs. This was the case for SMR as well as Gamma NF and for the real and sham feedback conditions. However, these increases were not statistically significant. Hence, the number of miniature saccades increased over the NF training course numerically but not specifically in a certain feedback condition or group.

### Relation Between NF Performance and Miniature Saccades

The results of the correlation analyses show the same picture. All correlations between the NF performance and the increase in the number of miniature saccades were positive but statistically not significant. This is a first sign that miniature saccades did not systematically affect NF training performance. In a further step we used the classification of participants based on their saccade frequencies (classification into saccade increasers and non-increasers) to predict their NF performance. Again, we found no systematic relation. Finally, we tested the original classification of NF responders and non-responders under the assumption that the miniature saccade rates for these groups were equally (i.e., randomly) distributed. We found that the observed saccade rates did not differ from this expectation, thus confirming the lack of a relation between NF performance and saccadic behaviour.

Despite these results it would be premature to rule out any impact of miniature saccades on NF training performance. The design of our study followed well established standards from previous and current NF research where a number of participants are being trained over a relatively short period of time or runs, and the resulting performance is compared against a sham control. Unlike typical experimental manipulations in vision research, for example, it cannot be assumed that the experimental treatment (i.e., the NF) exerts its effect on all participants in a similar way. Rather, it is typical for NF research that the treatment has an effect only in some participants but not in others. This was the case in our study where we found 58% and 38% responders, respectively. This selective responsiveness of participants poses a big challenge for the detection of variables such as eye movements that may co-vary with the treatment effects. If the effect of interest is absent in a larger percentage of participants it is difficult to detect confounds with such effects. An alternative approach would be to select only responsive individuals who are able to reliably increase the NF power and to investigate possible confounds with eye movements on the single subject level. The problem with this approach is, however, that strategies to maximize NF power may vary between individuals, and while some participants may change their eye movement behaviour, others might use other strategies to increase NF power. The challenge then is to identify individuals with altered eye movement behaviour. But even if this can be accomplished, such individuals may not be representative for the variety of strategies used in NF training (Autenrieth et al., 2020; Davelaar et al., 2018; Kober et al., 2013). As it stands, our investigation is the first one to explore the relation between NF performance and miniature saccades in a typical NF design, and we could not establish a systematic relationship. It is clear to us that more research is needed to definitively rule out the possibility that eye movements have no effect on NF performance.

### Design of Visual Feedback Displays in NF Studies

Most NF studies use relatively simple visual feedback designs, where for instance a two-dimensional object (a ball or a bar) increases and decreases in size (Enriquez-Geppert et al., 2017) similar to what we have used in the present study. However, there are also attempts to use a more realistic visual feedback, e.g., showing an animated hand changing its posture from open to a grasp (Ono et al., 2013), using virtual three-dimensional scenarios (Berger et al., 2022; Kober et al., 2016, 2017a), or using game-like feedback scenarios (Ninaus et al., 2014), to increase motivation and adherence to NF training. Such visual feedback modalities, which are visually richer than a simple moving bar, could also trigger more and larger saccades. It is a matter of future studies to investigate the effects of larger saccades in visually richer feedback displays on NF training performance. Here we used a relatively simple visual feedback design, which has often been used in previous NF training studies (Enriquez-Geppert et al., 2017; Kober et al., 2015, 2017b). Such simple visual feedback with moving objects might be a good compromise between information content and simplicity, which is relatively unaffected by eye movement artifacts.

### Limitations

Our study has some obvious limitations. The relatively small sample size limits the generalizability of our results. Future studies should investigate larger and more diverse samples, e.g., participants of different age groups as it is well known that oculomotor and perceptual performance declines with age (e.g., Dowiasch et al., 2015; Klein et al., 2000); this also holds for clinical populations (Terao et al., 2017). As NF training is particularly important for patients with neuropsychiatric conditions, such studies would greatly improve the generalizability of results beyond our sample of healthy young adults. Additionally, we only performed one NF training session. NF training performance and strategies to control the feedback signal might change over time (Domingos et al., 2021; Gruzelier, 2014; Kober et al., 2013). Therefore, it would be useful to investigate possible changes in eye movement behavior and the resulting effects on EEG activity and NF performance across several NF training sessions in the future.

## Conclusion and Implications

In the present study, we showed that miniature saccades make up the majority of saccades in our relatively simple visual feedback design. Our results have practical implications for the use of NF in clinical practice, where eye movements are hardly controlled or controllable. We could show that saccades indeed lead to an increase in EEG power in a broad Gamma frequency range. However, we did not find a systematic relation between miniature eye movements and NF training performance. Our results cannot rule out such a relationship and emphasize the need for further research.

### Supplementary Information

Below is the link to the electronic supplementary material.

Supplementary file1 (DOCX 1060 KB)

## Data Availability

Data that support the findings of this study are available on request from the corresponding author (S.E.K.) after contacting the Ethics Committee of the University of Graz (ethikkommission@uni-graz.at) for researchers who meet the criteria for access to confidential data. These ethical restrictions prohibit the authors from making the data set publicly available.
