# Supplementary material for: Do Miniature Eye Movements Affect Neurofeedback Training Performance? A Combined EEG-Eye Tracking Study
Source: Appl Psychophysiol Biofeedback. 2024 Mar 16;49(2):313–27. doi: 10.1007/s10484-024-09625-6 (PMC11101551; doi:10.1007/s10484-024-09625-6)
Supplement: Supplementary file 1 — Supplementary file1 (DOCX 1060 KB) [file 10484_2024_9625_MOESM1_ESM.docx]

**Supplementary Material**

**Title:** Do Miniature Eye Movements Affect Neurofeedback Training Performance? A Combined EEG-Eye Tracking Study

*Silvia Erika **Kober**^1,2^, Guilherme **Wood**^1,2^, Sarah **Schuster**^1^, Christof **Körner**^1,2^

^1^ Department of Psychology, University of Graz, Graz, Austria.

^2^ BioTechMed-Graz, Graz, Austria

Silvia Erika Kober (silvia.kober@uni-graz.at)

Guilherme Wood (guilherme.wood@uni-graz.at)

Sarah Schuster (sarah.schuster93@web.de)

Christof Körner (Christof.koerner@uni-graz.at)

***Correspondence:**

Dr. Silvia Erika Kober

Department of Psychology

University of Graz

Universitaetsplatz 2/III,

8010 Graz, Austria

Phone: +43 (0) 316 / 380 - 8497

Fax: +43 (0) 316 / 380 - 9808

E-Mail: silvia.kober@uni-graz.at

**Supplementary material A**

Description of questionnaires

The KUT questionnaire (German shortcut for “Kontrollueberzeugug im Umgang mit Technik” - Control beliefs in dealing with technology) assesses the locus of control of reinforcement (LOC) in the context of dealing with technology (Beier, 1999, 2004). Participants rate their actual technologic biography using 8 items on a five-point Likert scale (range of total score: 8-40). The questionnaire is available in German and has a Cronbach’s alpha of .89. The KUT was applied during both NF sessions (SMR and Gamma session).

To assess the motivation to perform the NF training a standardized motivation questionnaire, the Questionnaire on Current Motivation (Fragebogen zur Erfassung Aktueller Motivation, FAM, Rheinberg et al., 2001) adapted to the NF context (Kleih et al., 2010) was used before the SMR and Gamma NF training session. The FAM uses 18 items to measure four motivational factors in either field or laboratory learning and achievement situations: Incompetence Fear (anxiety), Mastery Confidence (probability of success), Interest, and Challenge. This questionnaire uses a seven-point Likert scale (range of total score per scale: 4-28).

Right after each NF task (real and sham SMR NF, real and sham Gamma NF), participants reported on their subjectively perceived level of concentration during NF training using a ten-point Likert-scale ranging from 1 (no control / very low concentration) to 10 (high control / high concentration). The two questions used were: Q1: “How focused were your thoughts on the neurofeedback task?”; Q2: “How well were you able to concentrate during the neurofeedback session compared to your other concentration?”

Additionally, after each NF task (real and sham SMR NF, real and sham Gamma NF) participants were asked to write down in their own words the mental strategies they have used to control the feedback bars. The reported mental strategies were qualitatively merged to different categories in line with previous studies investigating mental strategies during NF training (Kober et al., 2013; Autenrieth et al., 2020).

Questionnaire results

Table A1 summarizes questionnaire data of the KUT, the FAM, and the two questions about the level of concentration during NF. A series of *t*-tests revealed no significant differences between responders and non-responders (all *p* > 0.12). For the questions about the level of concentration during NF, also no significant differences between sham and real feedback conditions were observed (all *p* > 0.09).

The used mental strategies did not differ considerably between NF tasks (Figure A1 and A2). In line with prior studies, concentration strategies were mentioned most frequently (Kober et al., 2013; Kober et al., 2017; Autenrieth et al., 2020).

Table A1

*Means of raw values and SD of questionnaire data to assess motivation, control beliefs, and subjectively perceived level of concentration during NF training per NF task*

|  | SMR responder | SMR non-responder | Gamma responder | Gamma non-responder |
| --- | --- | --- | --- | --- |
|  | Mean (SD) | Mean (SD) | Mean (SD) | Mean (SD) |
| KUT | 23.57 (2.10) | 23.40 (2.80) | 23.88 (2.10) | 23.00 (2.42) |
| FAM - Mastery confidence | 22.14 (3.76) | 22.60 (4.01) | 21.13 (3.80) | 22.54 (2.63) |
| FAM -  Incompetence fear | 10.79 (4.42) | 10.10 (5.45) | 12.75 (6.30) | 10.31 (5.68) |
| FAM -  Interest | 25.57 (4.13) | 27.70 (5.64) | 27.13 (6.01) | 25.54 (5.14) |
| FAM -  Challenge | 20.64 (3.20) | 18.00 (5.01) | 20.75 (4.10) | 18.92 (3.52) |
| Concentration Q1 – real feedback | 7.74 (1.41) | 7.65 (2.55) | 6.43 (2.40) | 8.04 (2.10) |
| Concentration Q2 – real feedback | 7.21 (2.07) | 7.81 (2.02) | 6.76 (2.01) | 7.35 (2.54) |
| Concentration Q1 – sham feedback | 7.53 (1.98) | 7.58 (2.30) | 8.08 (1.63) | 8.02 (2.04) |
| Concentration Q2 – sham feedback | 7.16 (2.24) | 7.52 (2.19) | 7.85 (1.75) | 7.89 (2.27) |

*FAM = Questionnaire on Current Motivation (Fragebogen zur Erfassung Aktueller Motivation); KUT = Control beliefs in dealing with technology (Kontrollueberzeugug im Umgang mit Technik).*


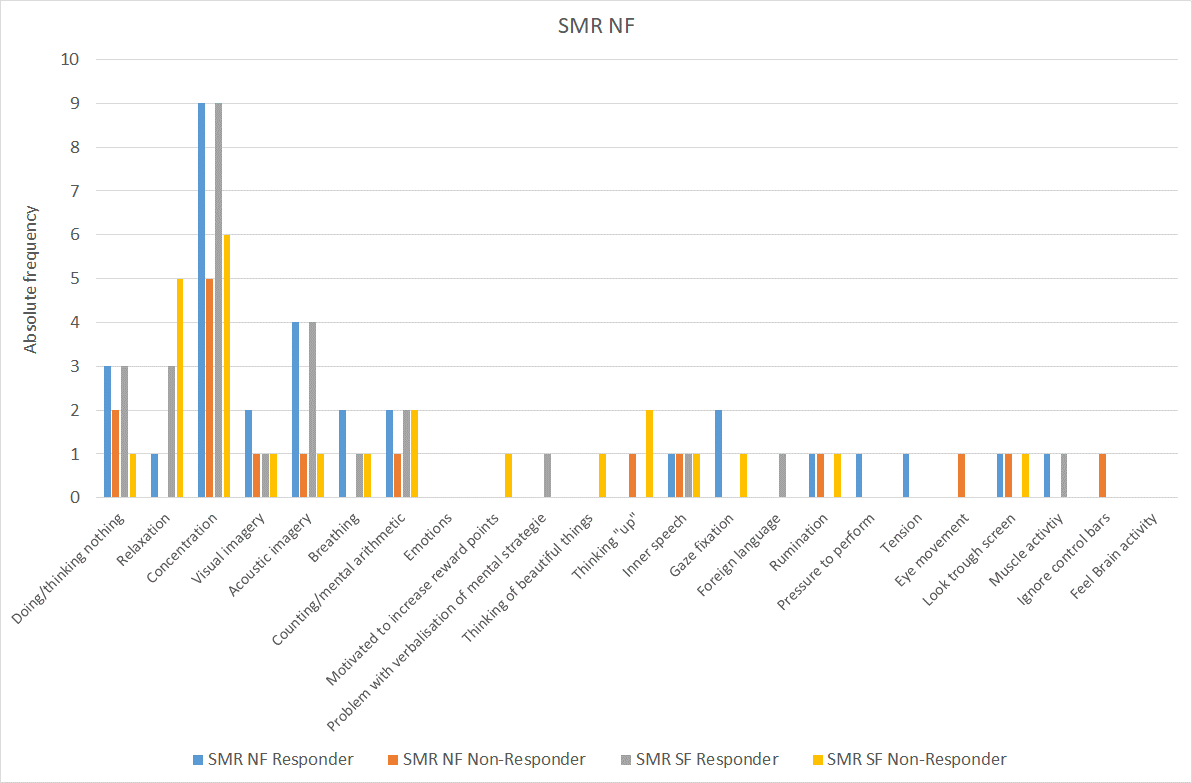


*Figure A1.* Number of individual reports (absolute frequencies) of mental strategies used during the SMR NF session, presented separately for responders and non-responders and the real and sham feedback condition.


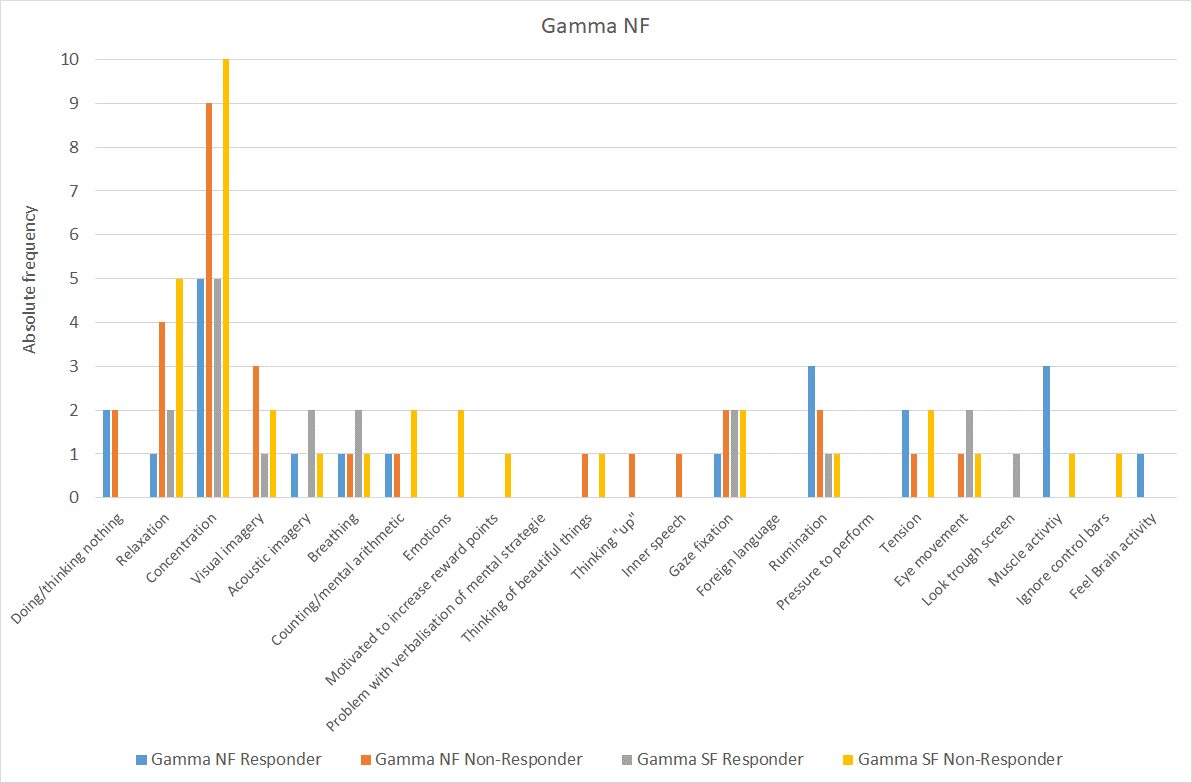


*Figure A2.* Number of individual reports (absolute frequencies) of mental strategies used during the Gamma NF session, presented separately for responders and non-responders and the real and sham feedback condition.

**Supplementary material B**

Table B1

*Consensus on the reporting and experimental design of clinical and cognitive-behavioral neurofeedback studies (CRED-nf checklist) best practices checklist (Ros et al., 2020)*

| **Domain** | **Item #** | **Checklist item** | **Reported in** | **Comments** |
| --- | --- | --- | --- | --- |
| **Pre-experiment** | | | |  |
|  | 1a | Pre-register experimental protocol and planned analyses | X | This was an explorative study. |
|  | 1b | Justify sample size | X | This was an explorative study, sample size was determined based on previous studies (Ninaus et al., 2015; e.g., Kober et al., 2017). |
| **Control groups** | | | |  |
|  | 2a | Employ control group(s) or control condition(s) | Methods section | Sham control condition, different feedback frequencies (SMR and Gamma) |
|  | 2b | When leveraging experimental designs where a double-blind is possible, use a double-blind | Methods section | Participants did not know whether they received real or sham feedback, Gamma or SMR feedback, experimenters did not know which participant received real feedback and who received sham feedback during NF training. |
|  | 2c | Blind those who rate the outcomes, and when possible, the statisticians involved | X |  |
|  | 2d | Examine to what extent participants and experimenters remain blinded | Methods section | Participants and experimenters were blinded during NF training concerning real/sham feedback conditions. |
|  | 2e | In clinical efficacy studies, employ a standard-of-care intervention group as a benchmark for improvement | X | This was no clinical efficacy study. |
| **Control measures** | | | |  |
|  | 3a | Collect data on psychosocial factors | Supplemental Material A |  |
|  | 3b | Report whether participants were provided with a strategy | Methods section |  |
|  | 3c | Report the strategies participants used | Supplemental Material A |  |
|  | 3d | Report methods used for online-data processing and artifact correction | Methods section |  |
|  | 3e | Report condition and group effects for artifacts | Results section & Supplemental Material C |  |
| **Feedback specifications** | | | |  |
|  | 4a | Report how the online-feature extraction was defined | Methods section |  |
|  | 4b | Report and justify the reinforcement schedule | Methods section |  |
|  | 4c | Report the feedback modality and content | Methods section |  |
|  | 4d | Collect and report all brain activity variable(s) and/or contrasts used for feedback, as displayed to experimental participants | Methods section & Results section & Supplemental Material C |  |
|  | 4e | Report the hardware and software used | Methods section |  |
| **Outcome measures** | | | |  |
| Brain | 5a | Report neurofeedback regulation success based on the feedback signal | Results section & Supplementary Material C |  |
|  | 5b | Plot within-session and between-session regulation blocks of feedback variable(s), as well as pre-to-post resting baselines or contrasts | Results section & Supplementary Material C | Only one NF training session was performed. Therefore, no between-session regulation can be plotted. |
|  | 5c | Statistically compare the experimental condition/group to the control condition(s)/group(s) (not only each group to baseline measures) | Results section  & Supplementary Material C |  |
| Behaviour | 6a | Include measures of clinical or behavioural significance, defined a priori, and describe whether they were reached | X | Only one NF training session was performed. |
|  | 6b | Run correlational analyses between regulation success and behavioural outcomes | X |  |
| **Data storage** | | |  |  |
|  | 7a | Upload all materials, analysis scripts, code, and raw data used for analyses, as well as final values, to an open access data repository, when feasible | See data availability |  |

**Supplementary material C**

To test whether the control EEG frequencies (4-7 Hz and 50-100 Hz) changed across the NF training, regression analyses were performed with feedback run number (6 feedback runs) as predictor variable and either 4-7 Hz or 50-100 Hz power as dependent variable. One-sample t-tests were calculated for each group (responder and non-responder) and condition (real/sham SMR and Gamma NF) to test whether the regression slopes differ from zero.

Analysis of power in 4-7 Hz frequency range (control frequency I)

Mean power values of control frequency I per group (responders vs. non-responders) and condition (real/sham SMR and Gamma NF) across NF runs can be seen in Figure C1.


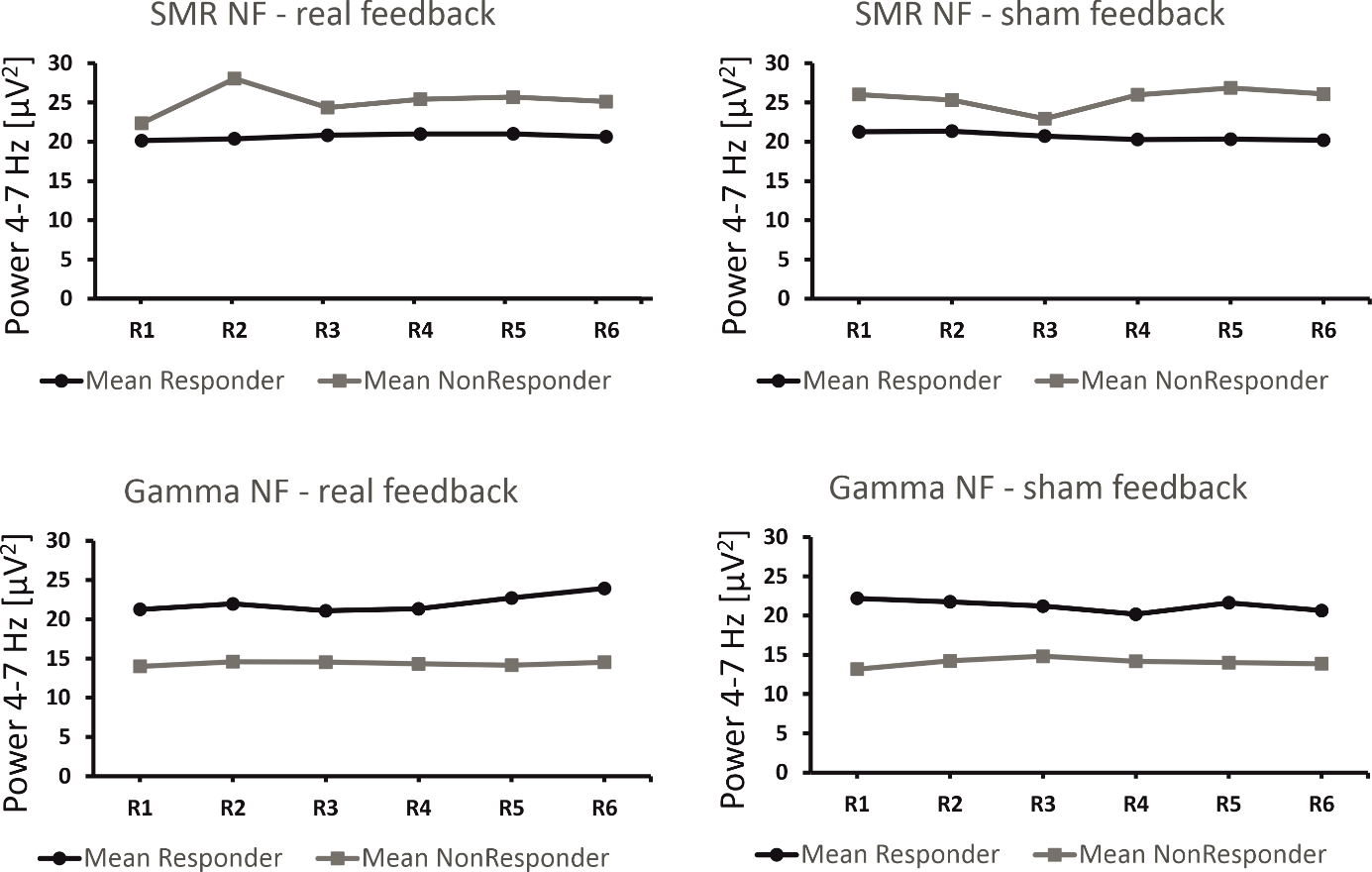


*Figure C1.* Changes in EEG control frequency I across feedback runs R1 – R6, presented separately for responders and non-responders and real/sham SMR/Gamma feedback.

Results of the *t*-tests against 0 can be found in Table C1. All results were not significant after Bonferroni correction.

Table C1

*Results of one-sample t-tests comparing regression slopes of control frequency I against 0*

|  | *df* | *t*-value | *p*-value |
| --- | --- | --- | --- |
| SMR NF real feedback – responders | 13 | 1.00 | 0.335 |
| SMR NF real feedback – non-responders | 9 | -0.89 | 0.396 |
| SMR NF sham feedback – responders | 13 | -2.44 | 0.030 |
| SMR NF sham feedback – non-responders | 9 | 0.09 | 0.927 |
| Gamma NF real feedback – responders | 7 | 1.17 | 0.279 |
| Gamma NF real feedback – non-responders | 12 | 0.29 | 0.778 |
| Gamma NF sham feedback – responders | 7 | -1.87 | 0.103 |
| Gamma NF sham feedback – non-responders | 12 | 0.85 | 0.413 |

* significant after Bonferroni correction

Analysis of power in 50-100 Hz frequency range (control frequency II)

Mean power values of control frequency I per group (responders vs. non-responders) and condition (real/sham SMR and Gamma NF) across NF runs can be seen in Figure C2.


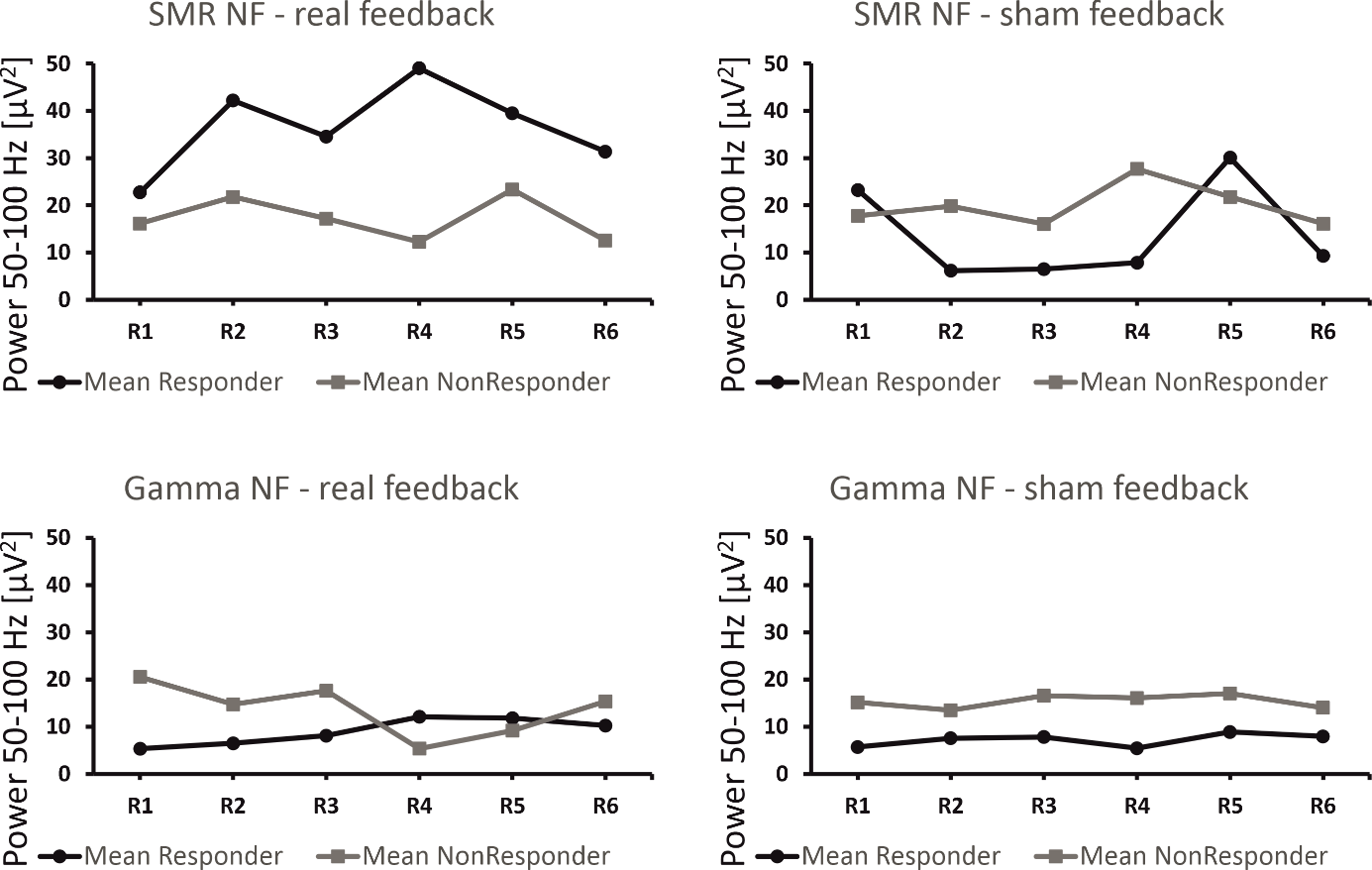


*Figure C2.* Changes in EEG control frequency II across feedback runs R1 – R6, presented separately for responders and non-responders and real/sham SMR/Gamma feedback.

Results of the *t*-tests against 0 can be found in Table C2. All results were not significant after Bonferroni correction.

Table C2

*Results of one-sample t-tests comparing regression slopes of control frequency II against 0*

|  | *df* | *t*-value | *p*-value |
| --- | --- | --- | --- |
| SMR NF real feedback – responders | 13 | 1.02 | 0.327 |
| SMR NF real feedback – non-responders | 9 | -0.82 | 0.434 |
| SMR NF sham feedback – responders | 13 | 0.08 | 0.936 |
| SMR NF sham feedback – non-responders | 9 | -0.20 | 0.846 |
| Gamma NF real feedback – responders | 7 | 2.45 | 0.044 |
| Gamma NF real feedback – non-responders | 12 | -1.62 | 0.131 |
| Gamma NF sham feedback – responders | 7 | 0.55 | 0.598 |
| Gamma NF sham feedback – non-responders | 12 | 0.42 | 0.684 |

* significant after Bonferroni correction

**Supplementary material D**

Number of saccades per second for different sizes of saccades. The majority of saccades were so-called ‘‘miniature saccades’’, which were defined as saccades smaller than 1⁰.


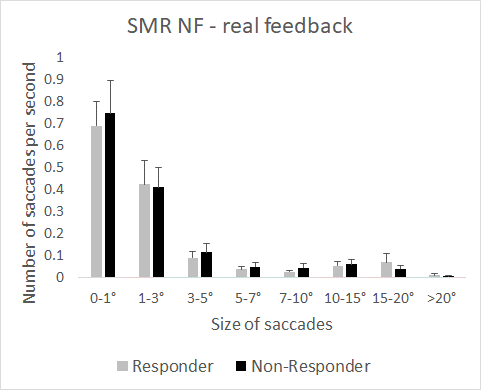

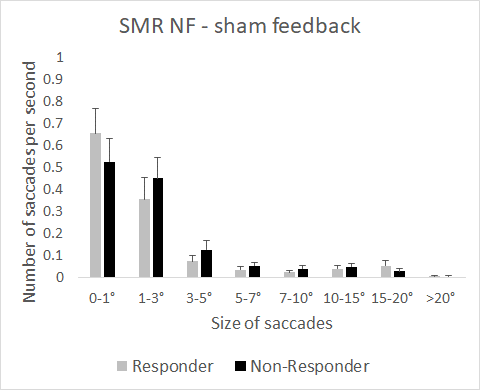

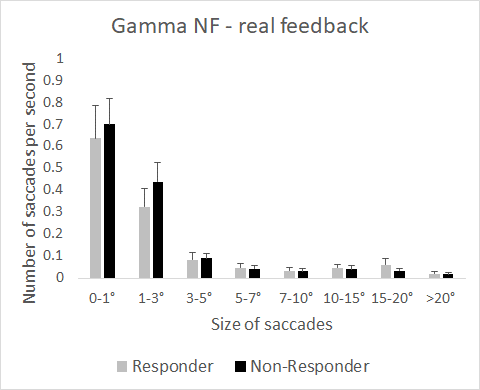

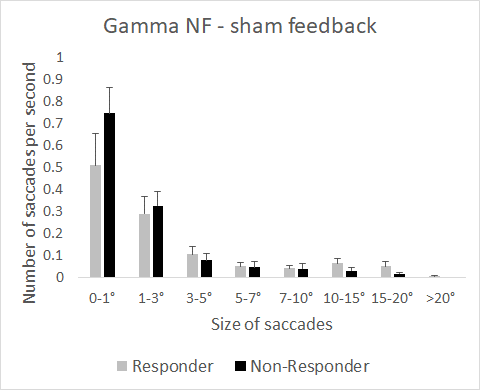


*Figure D1.* Number of saccades per second for different sizes of saccades.

Changes in the number of all saccades per second across the NF training can be seen in Figure D2.


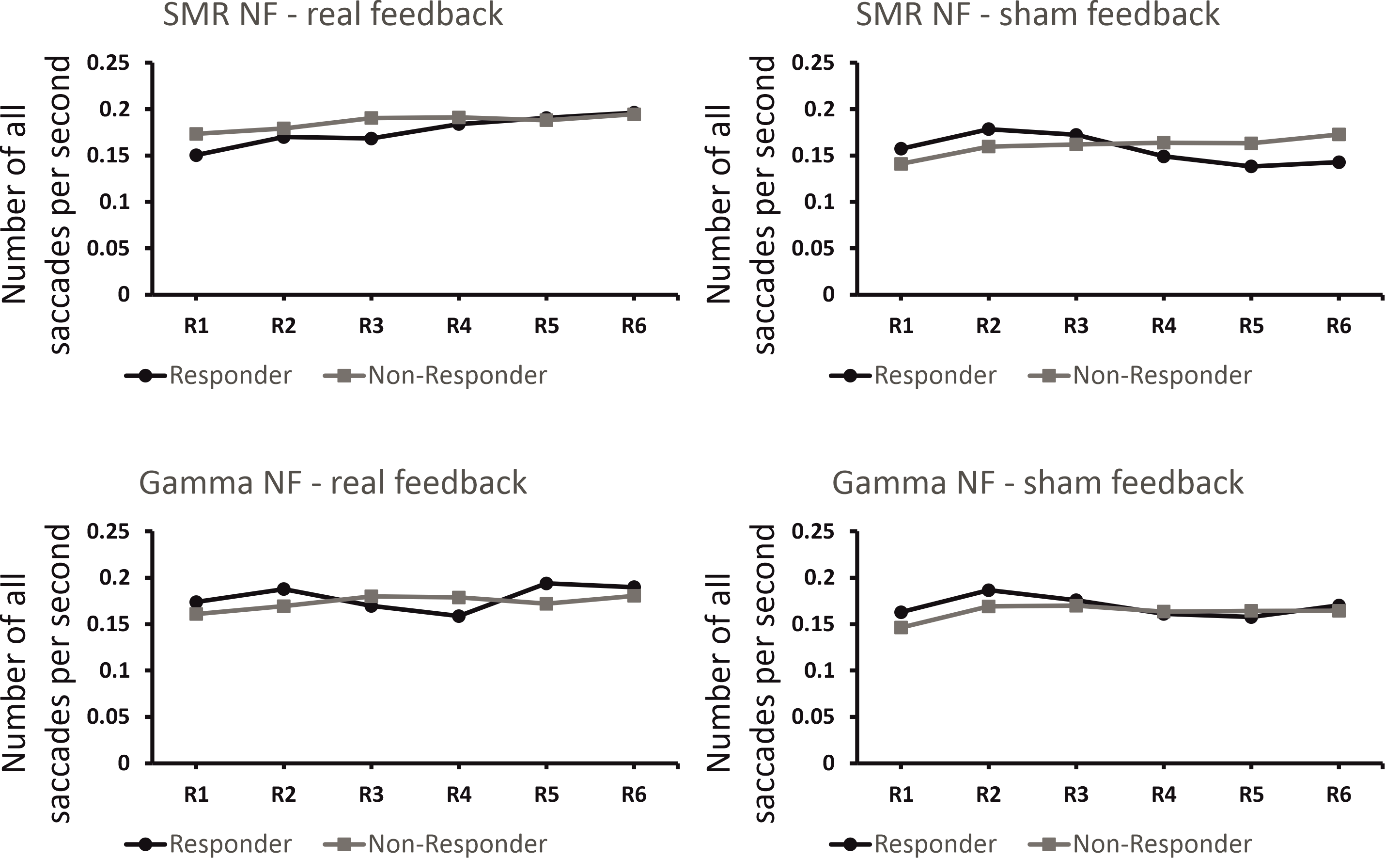


*Figure D2: Changes in the number of all saccades per second across feedback runs R1 – R6, presented separately for responders and non-responders and for the different conditions (SMR NF top panels, Gamma NF bottom panels; real feedback left panels, sham feedback right panels).*

REFERENCES

Autenrieth, M., Kober, S. E., Neuper, C., and Wood, G. (2020). How Much Do Strategy Reports Tell About the Outcomes of Neurofeedback Training? A Study on the Voluntary Up-Regulation of the Sensorimotor Rhythm. *Front. Hum. Neurosci* 14, 218. doi: 10.3389/fnhum.2020.00218

Beier, G. (1999). Kontrollüberzeugungen im Umgang mit Technik. *Report Psychologie* 9, 684–693.

Beier, G. (2004). *Kontrollüberzeugungen im Umgang mit Technik: Ein Persönlichkeitsmerkmal mit Relevanz für die Gestaltung technischer Systeme*. Doctoral thesis. Berlin.

Kleih, S., Nijboer, F., Halder, S., and Kübler, A. (2010). Motivation modulates the P300 amplitude during brain–computer interface use. *Clinical Neurophysiology* 121, 1023–1031. doi: 10.1016/j.clinph.2010.01.034

Kober, S. E., Witte, M., Ninaus, M., Koschutnig, K., Wiesen, D., Zaiser, G., et al. (2017). Ability to Gain Control Over One’s Own Brain Activity and its Relation to Spiritual Practice: A Multimodal Imaging Study. *Front. Hum. Neurosci* 11, 1–12. doi: 10.3389/fnhum.2017.00271

Kober, S. E., Witte, M., Ninaus, M., Neuper, C., and Wood, G. (2013). Learning to modulate one's own brain activity: the effect of spontaneous mental strategies. *Front. Hum. Neurosci* 7, 1–12. doi: 10.3389/fnhum.2013.00695

Ninaus, M., Kober, S., Witte, M., Koschutnig, K., Neuper, C., and Wood, G. (2015). Brain volumetry and self-regulation of brain activity relevant for neurofeedback. *Biological Psychology* 110, 126–133. doi: 10.1016/j.biopsycho.2015.07.009

Rheinberg, F., Vollmeyer, R., and Burns, B. D. (2001). FAM: Ein Fragebogen zur Erfassung aktuller Motivation in Lern- und Leistungssituationen. *Diagnostica* 47, 57–66. doi: 10.1026//0012-1924.47.2.57

Ros, T., Enriquez-Geppert, S., Zotev, V., Young, K. D., Wood, G., Whitfield-Gabrieli, S., et al. (2020). Consensus on the reporting and experimental design of clinical and cognitive-behavioural neurofeedback studies (CRED-nf checklist). *Brain* 143, 1674–1685. doi: 10.1093/brain/awaa009
